# Understanding *Toxoplasma gondii* transmission in an ecological context—the contribution of wild avian species from urban environments

**DOI:** 10.3389/fvets.2025.1634254

**Published:** 2025-09-04

**Authors:** Aleksandra Penezić, Aleksandra Uzelac, Katarina Breka, Stanislav Simin, Kristijan Ovari, Ilija Pantelić, Vladimir Ćirković, Duško Ćirović, Ivana Klun

**Affiliations:** ^1^Faculty of Biology, University of Belgrade, Belgrade, Serbia; ^2^Institute for Medical Research, University of Belgrade, Belgrade, Serbia; ^3^Department of Veterinary Medicine, Faculty of Agriculture, University of Novi Sad, Novi Sad, Serbia; ^4^Belgrade Zoo, Belgrade, Serbia

**Keywords:** synanthropic birds, backyard chicken, rodents, water, environment

## Abstract

**Introduction:**

The role of avians in the transmission chain of *Toxoplasma gondii*, a zoonotic coccidian parasite of the phylum Apicomplexa, is as intermediate hosts. However, the true contribution and significance of wild species in the maintenance and spread of the parasite in different ecosystems is not well understood.

**Methods:**

For this study, heart tissue of 224 individual birds, representing 15 common wild species, and one domestic, *Gallus gallus domesticus* (backyard chickens), was collected. Total nucleic acids were extracted and the presence of *T. gondii* gDNA was ascertained by amplification of the 529 bp repeat element.

**Results:**

The infection was detected in 24.1% of the wild birds and in 41.4% of backyard chickens. The occurrence of infection in wild species did not statistically differ by diet or among urban (22.4%), peri-urban (27.3%) and rural areas (22.7%); in contrast, a statistically significant difference was observed between peri-urban (21%) and rural (80%) backyard chickens. Among the 11 city dwelling species, wood pigeons (*Columba palumbus*), rooks (*Corvus frugilegus*) and hooded crows (*Corvus cornix*) were the most numerous. The frequency of infection in the two corvid species was 32.1% and 31.6% in rooks and hooded crows, respectively, and 15.6% in wood pigeons, suggesting that corvids may be good bioindicators for the parasite in cities. As the majority (84%) of the city dwelling birds originated from a single residential area, possible local natural reservoirs of *T. gondii*, rodents and water, were analyzed additionally. Of the 16 rodents, 56.2% were infected, while three out of four samples of river water harbored *T. gondii* gDNA, indicating a fairly high probability of exposure to the parasite.

**Discussion:**

Collectively, our findings show that diet may not be a primary risk for *T. gondii* infection. Instead, the importance of understanding prevalence in birds in an ecological context and the contribution of environmental factors in different habitats are highlighted.

## 1 Introduction

Many avian species are common residents of cities and suburban environments in Europe. Domestic chicken, which are predominantly kept at rural farms, can also be encountered in suburban backyards of cities worldwide. This may be due to an existing tradition of backyard farming in some countries, while more recently, the trend of keeping chickens by private individuals was inspired by the popularity of organic/free-range eggs and meat (1–3). Ornamental duck species, such as the mandarin and wood duck (*Aix galericulata* and *Aix sponsa*), have been purposely introduced in European parks and estates with suitable freshwater habitats. While the mandarin duck was not endemic when it was first introduced, it has managed to establish breeding populations in the Netherlands and has been sighted in the wild as far south as Algeria recently (4, 5). The close proximity of non-endemic to endemic avians, both wild and domestic, facilitates the possibility of physical interaction and provides opportunity for pathogen transmission. Birds raised extensively or in backyards are especially vulnerable, as confinement and isolation are usually not practiced. One of the greatest global health challenges in the 21^st^ century thus far has been the emergence of ‘new’ diseases, often zoonoses originating from wildlife, along with increased geographical spread of diseases (2, 6). Birds can transmit a variety of pathogenic bacteria, viruses and parasites to other birds, but also to mammals, including humans, thus playing a role in various transmission chains. Some of the most notorious zoonotic pathogens directly transmitted by birds to humans via ingestion or inhalation are the bacteria *Salmonella typhimurium*, *Campylobacter jejuni*, the Influenza A viruses, as well as *Cryptococcus neoformans*, a pathogenic fungus and the Apicomplexan parasites *Cryptosporidium meleagridis* and *Toxoplasma gondii*. Of these, only cryptococcosis may be considered an emerging disease, associated with immunodeficiencies (HIV), transplantation and immunosuppressive therapy (7, 8), while the other pathogens are responsible for ‘old’ diseases. Systematic active surveillance of bird reservoirs is in place only for the bacterial pathogens and avian influenza, while detection of *C. neoformans*, *C. meleagridis* and *T. gondii* occurs primarily through scientific investigations. Of the three, infection with *T. gondii* is associated with the highest disability adjusted life-years (DALY) (9). *T. gondii* infections occur globally with variable prevalence in humans and animals. The mode of infection is always ingestion, and as humans, animals and even the environment are reservoirs of the parasite, epidemiological research on *T. gondii* requires a One Health approach. The parasite’s life cycle is complex with any warm-blooded species acting as intermediate hosts and members of the family Felidae as definitive hosts. The role of cold-blooded hosts in the life cycle of the parasite is still unclear, despite the apparent success of short-term replication of *T. gondii* tachyzoites in some cold-blooded species after experimental infection and detection of parasite DNA in others (10, 11). There are three infectious life forms of *T. gondii*, the free living tachyzoite and two encysted forms: the bradyzoite (in tissue cysts) and the sporozoite (in oocysts) (12). The parasite can switch between tachyzoite and bradyzoite interchangeably in all hosts, but only in the definitive hosts can tachyzoites differentiate into intermediates required for sexual reproduction, which results in the formation of oocysts (13). Oocysts are expelled into the environment from the digestive tract of the definitive host where they undergo a process of maturation, under permissive environmental conditions, to yield sporocysts with sporozoites. Sporocysts are characterized by high tolerance of environmental conditions, with the capacity to remain infectious for extended periods of time in soil of various chemical compositions and also in fresh and marine water (14–16). *T. gondii* infection can occur after ingestion of tissue cysts in undercooked or raw meat, oocysts in mollusks, or on vegetables and fruits, as well as oocysts from untreated water, commonly from surface sources. Host to host transmission occurs dominantly via predation and on occasion vertically, while the frequency of environmental transmission is likely variable, depending on a multitude of factors (17). Avian species, similar to mammals, are exposed to multiple sources of infection, due to a highly variable diet and feeding habits. As with mammals, susceptibility to toxoplasmosis varies between avian species, thus, while domestic chicken handle the infection quite well (18), for others it can be lethal, even leading to near extinction in extreme cases, as with the ‘alalā (*Corvus hawaiiensis*) (19). High susceptibility to infection can reduce the host’s efficiency to transmit the parasite and vice versa, thus the capacity to maintain the parasite in the ecosystem differs by host biodiversity and population. In terms of spreading the parasite, unlike terrestrial species, the ability to fly allows birds to overcome a number of natural and anthropogenic environmental barriers, while migratory species are an exceptional category due to the additional ability to traverse vast distances over land and water. Thus, birds may be instrumental in disseminating and introducing *T. gondii* to remote, inaccessible and isolated ecosystems. A recent review and meta-analysis calculated the pooled global prevalence of *T. gondii* infection in birds to be 25% (23–28%), and 9% (4–17%), using serological and molecular detection methods, respectively (20). The lowest pooled prevalence (by all methods combined) of 16% (12–20%) was determined in Europe. Another study, by Wilson et al. (21), which examined seroprevalence in 24,344 wild birds from all continents except Australia, determined that omnivorous terrestrial species had the highest seroprevalence, while strictly carnivorous species came in second. In addition, the seroprevalence was greater in terrestrial as compared to aquatic species, while dietary preferences of aquatic species had a negligible impact on seroprevalence (21). The aim of this study was to assess the occurrence of *T. gondii* infection in different species of birds living in habitats with varying degrees of urbanization and human population density. Additionally, possible environmental (water) and animal reservoirs of infection relevant for birds were examined for the presence of parasite gDNA.

## 2 Materials and methods

### 2.1 Study design

To analyze the presence of *T. gondii* in different avian species and identify possible local animal and environmental reservoirs of infection for birds, parasite gDNA was detected by real-time PCR in heart tissues of birds and rodents as well as in filtered surface water.

### 2.2 Sample collection

The samples used in this study were collected in collaboration with hunting associations, avian rescue/rehabilitation centers and local poultry farmers. Hearts of domestic backyard chickens (*Gallus gallus domesticus*, *n* = 29) slaughtered for commercial use in 2024 were obtained from local farmers. All game bird species were collected during legal hunting seasons 2023 and 2024 and carcasses were provided by local hunters. Hearts of *Phasianus colchicus* (*n* = 45), *Anas platyrhynchos* (*n* = 35), *Corvus cornix* (*n* = 19), *Corvus frugilegus* (*n* = 28), *Columba palumbus* (*n* = 32), and *Columba livia domestica* (*n* = 6) were removed from the collected carcasses in a designated necropsy laboratory space. Carcasses of birds which had to be euthanized and/or succumbed to injury and/or malnourishment in a local avian rescue center during 2023 and 2024 were donated to the study by the management. The samples included *Ciconia ciconia* (*n* = 10), *Asio otus* (*n* = 8), *Otus scops* (*n* = 3), *Athene noctua* (*n* = 1), *Tyto alba* (*n* = 1), *Buteo buteo* (*n* = 4), *Accipiter nisus* (*n* = 1), *Accipiter gentilis* (*n* = 1), and *Falco tinnunculus* (*n* = 1). GPS coordinates of the locations from which game bird samples and chicken hearts were obtained were recorded, while the sanctuary provided information on the approximate retrieval location of the rescued birds, if known. Surface water (river) samples and carcasses of rodents were collected in the municipality of Surčin, located in the western part of Belgrade between the Sava and Danube rivers. River water (10 L) was sampled at convenient locations with public access (*n* = 4), while rodent carcasses (*n* = 16, all mice, different species) were provided by licensed exterminators in the area. All sampling was done based on availability and convenience due to challenges such as (a) various levels of governmental protection, (b) sampling restrictions with respect to habitat protection, (c) low population size, and (d) the nature of backyard farming and availability of tissues of few individual birds per farm.

### 2.3 Sampling area

The majority of analyzed birds (*n* = 192) originated from the Belgrade metropolitan area located in central Serbia between the Danube and Sava rivers, which covers a territory of 3,234 km^2^ and has 1,683,229 registered residents, thus yielding a population density of approximately 520 residents per km^2^ (22). The urban core of the city, which lies at the confluence of the two rivers, covers a territory of 360 km^2^ and with a population of 1,383,875 residents (82.3% of the metropolitan area population), has the highest population density in Serbia of 3,844 residents per km^2^. Of the analyzed birds, 40% originated from the municipality of Surčin, which is located west of the urban core of Belgrade, with its northern border represented by the Danube and southern by the Sava River. Surčin covers a territory of 288 km^2^ with a registered population of 45,817, which represents roughly 2.7% of the entire metropolitan area population and yields a density of approximately 160 residents per km^2^. The area is primarily residential, with few two to four story buildings and mostly single-family homes surrounded by agricultural plots and small farms with gardens/orchards.

### 2.4 Ecology and wildlife habitats in the sampling area

Surčin, which is situated in the southern part of the Pannonian Plain, encompasses a diverse array of habitats that support a rich avian biodiversity. These habitats include river channels, fishponds, forests, and anthropogenic landscapes, each providing unique ecological niches for various bird species. The river channels serve as vital ecosystems for numerous bird species, supporting a variety of wetland birds, including herons, egrets, and waterfowl, which rely on the abundant aquatic resources for feeding and nesting. Wetlands are particularly crucial during migratory periods, offering essential stopover sites for birds traveling along the East–West migratory flyway. Within the Surčin municipality, the fishponds of Živača and Bečmen are well known habitats for wetland avifauna. These artificial water bodies mimic natural wetlands and provide critical resources for nesting and foraging. Human-modified landscapes, including agricultural fields, urban areas, and small farms, play a significant role in supporting urban bird populations as well as synanthropic bird species. Species such as the hooded crow (*Corvus cornix*), rook (*Corvus frugilegus*), and common starling (*Sturnus vulgaris*) thrive in these environments, utilizing tall trees, roadsides, and cultivated lands for nesting and foraging. These adaptable species have successfully integrated into human-dominated landscapes, contributing to the overall avian diversity of the region. Several designated hunting grounds, including “Donji Srem,” “Dobanovački zabran,” and “Crni lug” are integrated into the landscape. The habitats within hunting grounds support game species such as wild boar (*Sus scrofa*), roe deer (*Capreolus capreolus*), and pheasants (*Phasianus colchicus*). While primarily focused on game management, these hunting grounds also provide habitats for various bird species.

### 2.5 DNA extraction and qPCR

#### 2.5.1 Birds and rodents

The apex of the heart was excised, cut into several pieces and transferred into 2.0 mL microtubes containing ceramic beads of 1.4 mm diameter. Trizol reagent (Life Technologies, Carlsbad, CA, United States) was added to each tube and the tissue was homogenized using a bead mill, Bead Ruptor 4 (Omni International, Kennesaw, GA, United States). Genomic DNA was extracted from the homogenate according to the manufacturer’s instructions. Screening for the presence of *T. gondii* gDNA was performed by amplification of the 529 bp repeat element by qPCR as previously described (23). Each reaction contained 10 μL TaqMan Universal PC R Mastermix (Applied Biosystems, Foster City, CA, United States), 0.25 mM forward (F) and reverse (R) primers (5′-AGA GAC ACC GGA ATG CGA TCT-3′; 3′-CCC TCT CCA CTC TTC AAT TCT-5′), 0.10 mM of the specific TaqMan probe FAM-ACG CTT TCC TCG TGG TGA TGG CG-TAMRA (Invitrogen, Life Technologies, Carlsbad, CA, United States) and 3 μL of extracted gDNA in a final volume was 20 μL. The thermal cycling program included a 5 min initial denaturation at 95°C, followed by 45 cycles of denaturation at 95°C for 15 s and annealing/extension at 60°C for 60 s in a StepOnePlus Real Time PCR System (Applied Biosystems, Foster City, CA, United States). Each run included a positive control (gDNA of the RH strain of *T. gondii*) as well as a negative control (RNase/DNase free water).

#### 2.5.2 Water

River water was collected just below the surface, at a depth of up to 30 cm to avoid floating debris. The water was filtered using a peristaltic pump (ISI 10, AxFlow, Dublin, Ireland) through a membrane filter of 1.2 μm pore size (Millipore, Burlington, MA, United States) fixed inside a filter holder (Millipore, Burlington, MA, United States) (24). The pore size of the membrane is sufficiently small to trap *T. gondii* oocysts. The filter membrane was rinsed several times during the filtration process with a phosphate and detergent based wash buffer [8% NaCl, 0.2% KH_2_PO_4_, 2.9% Na_2_HPO_4_ (12H_2_O), 0.2% KCl, 1% SDS, 1% Tween-80, 0.1% Antifoam A] using a squirt bottle and finally transferred into a dish, submerged in wash buffer and vigorously manually scrubbed to dislodge trapped oocysts. The wash buffer was collected into 50 mL conical tubes and centrifuged at 3000 × *g* for 10 min in a swinging bucket desktop centrifuge (Heraeus Megafuge 1.0R, Kendro, Langenselbold, Germany). The supernatant was discarded, while the pellet was homogenized inside a bead mill and subsequently used for DNA extraction as described in 2.5.1.

### 2.6 Mapping and statistical analyses

The sample map was created using QGIS 3.40.2.^1^ Statistical differences in *T. gondii* occurrence between species or groups were determined by chi-square analysis, or Fisher’s exact test (where necessary). The level of significance was 5%. The analyses were performed using the SPSS v11.5 statistical package (SPSS Inc., Chicago, IL, United States).

## 3 Results

### 3.1 Bird samples

**Figure 1:**
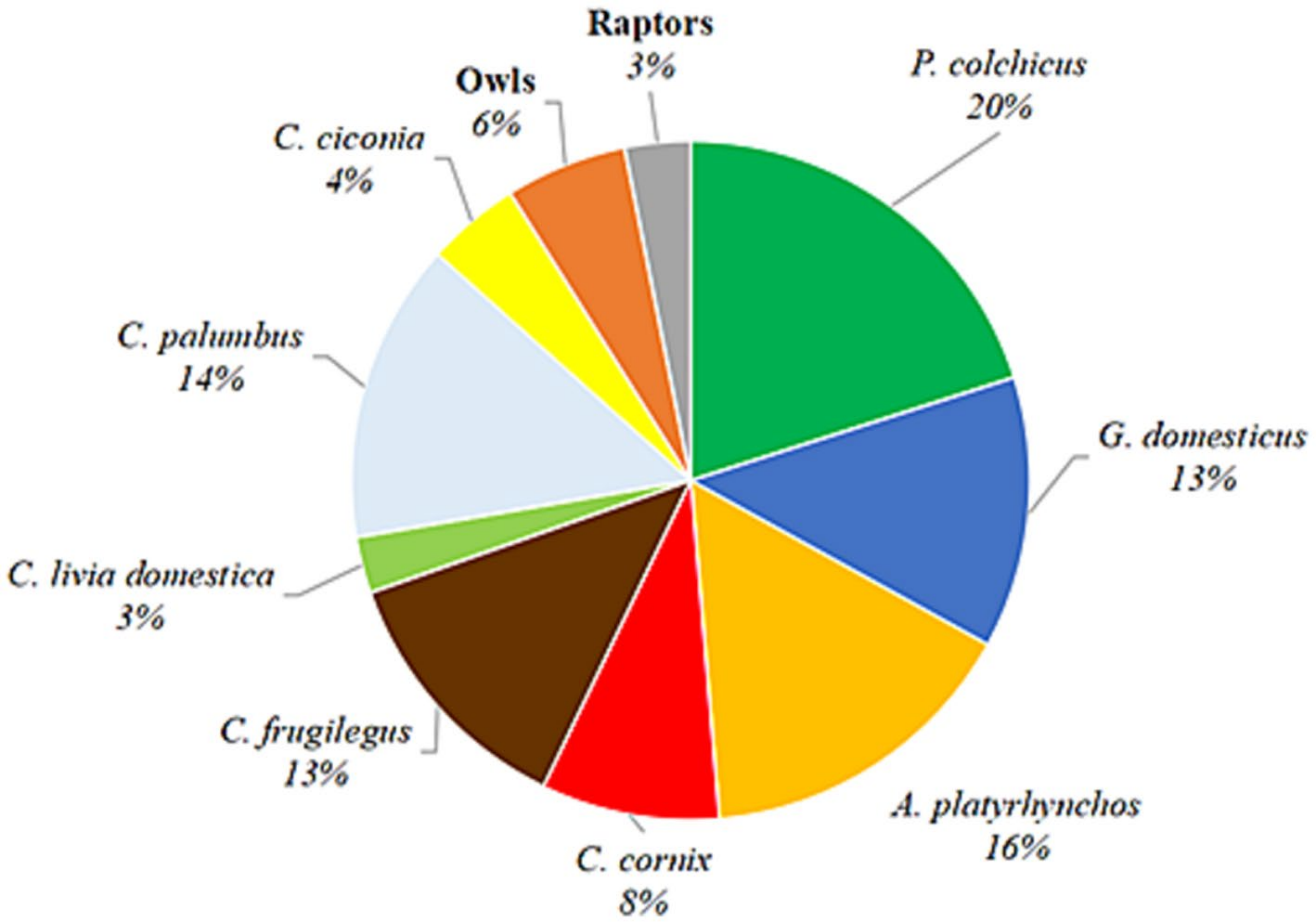
Pie chart of the bird species analyzed. The collected/obtained samples of different species are represented by the pie slices. Percentages are calculated from the total *n* = 224.

**Figure 2:**
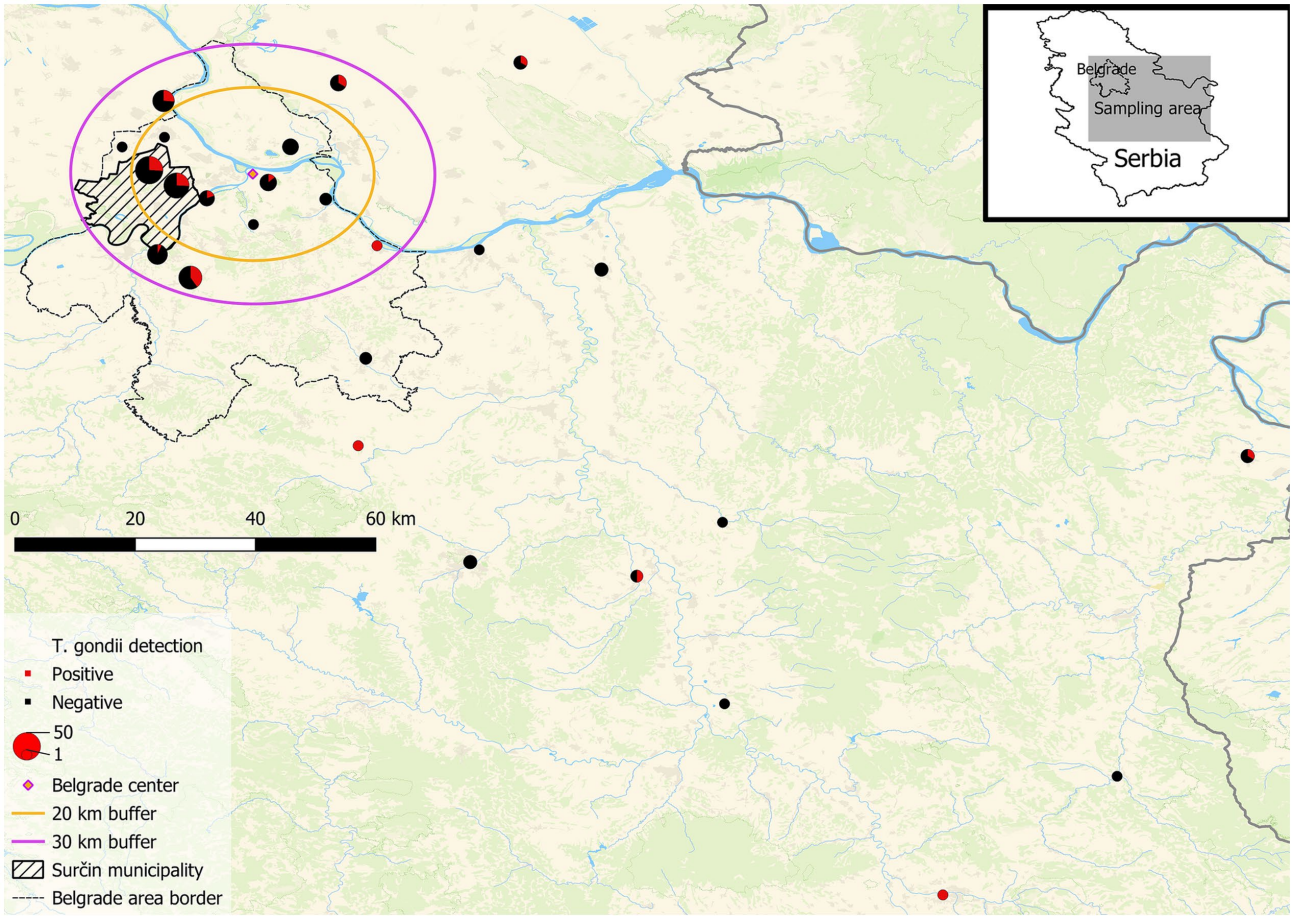
Map of the geographical origin of the bird samples. Map showing the sampling area encompassing part of central and most of eastern Serbia with Belgrade (the capital) located in the center of the concentric circles. Danube (E → W) is the largest river on the map. The radius of the inner circle is <20 aerial kilometers from the center of Belgrade (urban), the outer circle’s radius is at 30 aerial kilometers from the center (peri-urban), while the area outside the outer circle is mostly rural (significantly smaller built-up areas and lower population density as compared to Belgrade). The legend is shown in the lower left corner of the map.

In this study, samples of birds of nine avian families (Phasianidae, Corvidae, Columbidae, Anatidae, Ciconiidae, Strigidae, Accipitridae, Tytonidae, and Falconidae) were collected and analyzed for the presence of *T. gondii* gDNA in heart tissues (*n* = 224). In total, 16 different species are represented, 15 terrestrial and one aquatic, with different diet and feeding habits (Figure 1). The only domestic species analyzed were backyard chickens, whereas the remaining were game birds, synanthropic and other wild avian species. The greatest number of individual samples of a single species were pheasants (45/224), while the fewest number were feral pigeons (6/224). For purposes of analysis, four different species of owls (*A. otus*, *O. scops, A. noctua,* and *T. alba*) and of raptors (*B. buteo*, *A. nisus*, *A. gentilis,* and *F. tinnunculus*) were pooled into single categories, owls and raptors, respectively, due to low numbers of individuals. The precise or approximate location was available for all samples (Figure 2). The majority of birds originated from Serbia’s capital city of Belgrade and its peri-urban areas, while the remainder was from rural areas. Species used for human consumption, pheasants, chicken and ducks, represent 49% of the samples analyzed. Rooks, hooded crows, feral and wood pigeons, which are common in urban and rural environments and associate into large groups, make up 38% of the samples, while storks, owls and raptors which are less frequent in cities and associate into smaller groups, or live in pairs represent 13% of the samples (Table 1). Of all the species analyzed, only owls are nocturnal. In terms of the species’ diet, it can be roughly classified as dominantly grain/seed (Phasianidae and Columbidae), omnivorous and/or meat (Corvidae, owls and raptors), invertebrates/algae (Anatidae) and fish/amphibians (Cicconidae).

**Table 1:** Overview of *T. gondii* infection in birds (*n* = 224).

Species	No. infected/no. examined (%)	Description; feeding habits
*Phasianus colchicus* (pheasant)	13/45 (28.9)	Edible species; seed/grain
*Anas platyrhynchos* (mallard)	8/35 (22.9)	Edible species; invertebrates, algae
*Corvus cornix* (hooded crow)	6/19 (31.6)	Synanthropic, large groups; meat, omnivorous
*Corvus frugilegus* (rook)	9/28 (32.1)	Synanthropic, large groups; meat, omnivorous
*Columba livia domestica* (feral pigeon)	1/6 (16.7)	Synanthropic, large groups; seed/grain
*Columba palumbus* (wood pigeon)	5/32 (15.6)	Synanthropic, large groups; seed/grain
*Ciconia ciconia* (white stork)	1/10 (10)	Mostly rural, smaller groups; fish, amphibians
Owls*	1/13 (7.7)	Mostly rural, smaller groups; meat
Raptors**	3/7 (42.9)	Mostly rural, smaller groups; meat
Total wild birds	47/195 (24.1)	
*Gallus gallus domesticus* (domestic chicken)	12/29 (41.4)	Backyard husbandry, edible species; seed/grain

* Four species, Asio otus (*n* = 8), Otus scops (*n* = 3), Athene noctua (*n* = 1), and Tyto alba (*n* = 1). ** Four species, Buteo buteo (*n* = 4), Accipiter nisus (*n* = 1), Accipiter gentilis (*n* = 1), and Falco tinnunculus (*n* = 1).

### 3.2 *T. gondii* occurrence in birds

Parasite gDNA was detected in 26.3% of all samples analyzed, most frequently (per a single species) in heart tissue of domestic chickens (41.1%) and least frequently in white storks (10%) (Table 1). *T. gondii* gDNA was detected in 30.3% of the pheasants, mallards, and chicken (*n* = 109), which are used for human consumption, and in 22.6% of the remaining wild species (*n* = 115). The occurrence of infection based on dietary preferences was 27.7% in primarily seed/grain (*n* = 112), 28.3% in meat/omnivorous (*n* = 67) and 20% in fish/amphibian combined with invertebrate/algae (*n* = 45) (Table 1). To explore the potential impact of the degree of urbanization and human population density on the occurrence of *T. gondii* in birds, the wild avian samples were grouped based on the aerial distance from the center of Belgrade (the most urbanized area with the highest population density in all of Serbia), into a ‘urban’ (< 20 km) group with *n* = 107 samples, ‘peri-urban’ group (20–30 km) with *n* = 66 samples and a ‘rural’ group (>30 km) with *n* = 22 samples (Table 2). *T. gondii* infection was detected in 22.4% of the urban group, 27.3% of the peri-urban group and 22.7% of the rural group. As backyard chicken hearts were collected from peri-urban and rural environments, the occurrence of infection was analyzed and found to be 21 and 80%, respectively (Table 3). All of the results of the statistical comparisons performed are shown in Table 4.

**Table 2:** Impact of distance from highly urbanized areas and population density gradient on *T. gondii* infection of wild avian species (*n* = 195).

Classification	Species	*n*	*T. gondii* infection (%)	Overall infection in group (%)
Urban group (<20 aerial kilometer distance from Belgrade center)	*P. colchicus*	1	0	22.4
	*A. platyrhynchos*	16	18.7	
	*C. cornix*	19	31.6	
	*C. frugilegus*	28	32.1	
	*C. palumbus*	32	15.6	
	*C. ciconia*	3	0	
	Owls (*A. otus, O. scops, A. noctua*)	6	0	
	Raptors (*A. gentilis, F. tinnunculus*)	2	50	
	Total	107		
Peri-urban group (20–30 aerial kilometer distance from Belgrade center)	*P. colchicus*	40	30	27.3
	*A. platyrhynchos*	19	26.3	
	*C. livia domestica*	6	16.6	
	Owl (*O. scops*)	1	0	
	Total	66		
Rural group (>30 aerial kilometer distance from Belgrade center)	*C. ciconia*	7	14.3	22.7
	Owls (*A. otus, O. scops, T. alba*)	6	16.6	
	Raptors *(B. buteo, A. nisus)*	5	40	
	*P. colchicus*	4	25	
	Total	22		

**Table 3:** Impact of microenvironment (backyard) on *T. gondii* infection in chickens (*n* = 29).

Classification	Species	*n*	*T. gondii* infection (%)
Peri-urban backyard	*G. gallus domesticus*	19	21
Rural backyard		10	80

**Table 4:** Statistical analyses, chi-square, and Fisher *p*-value reported for backyard chicken.

Parameters	Test result	*p*-value
Comparison between species used in human diet and wild birds		
Phasianidae, Anatidae vs. combined other species	*χ*^2^ (1, *n* = 224) = 1.7	0.193
Comparisons based on diet (all birds)		
Seed/grain vs. meat/omnivorous	*χ*^2^ (1, *n* = 179) = 0.01	0.922
Seed/grain vs. fish/amphibian and algae/invertebrate	*χ*^2^ (1, *n* = 157) = 1.0	0.318
Meat/omnivorous vs. fish/amphibian and Algae/invertebrate	*χ*^2^ (1, *n* = 112) = 1.0	0.316
Comparisons based on distance from city center: wild species		
Urban vs. peri-urban	*χ*^2^ (1, *n* = 173) = 0.52	0.470
Urban vs. rural	*χ*^2^ (1, *n* = 129) = 0	0.976
Peri-urban vs. rural	*χ*^2^ (1, *n* = 88) = 0.18	0.674
Comparisons based on distance from city center: backyard chickens		
Peri-urban vs. rural	Fisher’s exact test (1, *n* = 29; two-tailed)	0.004

### 3.3 *T. gondii* in animal and environmental reservoirs in Surčin

As 84% of the birds (wood pigeons, *n* = 32; rooks, *n* = 28; hooded crows, *n* = 19, mallards, *n* = 10 and long eared owls, *n* = 1) from the urban group originated from one residential neighborhood of Belgrade (Surčin area), possible animal and environmental reservoirs of *T. gondii*, rodents and water, were analyzed from that area. The overall occurrence of *T. gondii* infection in all birds from Surčin was 25.8%, with very similar values for the two corvid species, rooks, 32.1% and hooded crows, 31.6, and 15.6% in wood pigeons. Of the 10 individual mallards, three were infected with *T. gondii*. Furthermore, *T. gondii* infection was present in 56.2% of mice (*n* = 16) while *T. gondii* gDNA was detected in three out of four water samples.

## 4 Discussion

This is the first report on the molecular occurrence of *T. gondii* infection in wild birds in Serbia which was determined to be 24.1%, nearly threefold higher than the average pooled global molecular prevalence in birds in the last two decades (20). Of the 16 species analyzed (Figure 1), the only domestic species were backyard chickens, which also had the highest occurrence of infection, 41.4% for a single species, while in wild species it was in rooks, 32.1% (Table 1). Previously, we determined seroprevalence of infection by modified agglutination assay (MAT) in pheasants (66.6%) and city dwelling feral pigeons (7.1%) (unpublished data). While it is not possible to directly compare sero- and molecular prevalence, due to fundamentally different approaches to detection—indirect (antibodies in sera or meat juice by various tests) and direct (PCR based detection of the parasite gDNA), the results suggest that infection is more frequent in pheasants as compared to feral pigeons, which was recapitulated by this study. Unlike the findings of Wilson et al. (21), based on a large global set of avian species and individuals, there were no statistical differences in the occurrence of infection based on diet or between terrestrial versus aquatic species (Table 4). However, this study found a higher molecular occurrence of infection in birds that are omnivorous (Corvidae, both species taken together, *n* = 47) as compared to strict carnivores (owls and raptors, combined, *n* = 20) with 31.9 and 20%, respectively. In recent years, several studies were published on the prevalence of *T. gondii* in wild bird species in Europe, mostly from southern Europe. The greatest number of individuals analyzed were of *Falco tinnuculus* (Eurasian kestrel) in Italy (*n* = 167 in 2016, *n* = 91 in 2017), with seroprevalences of 33.3 and 14.3% in 2016 and 2017, respectively (25), followed by *Columba livia* (feral pigeons) from Spanish zoos (*n* = 142), with a seroprevalence of 9.2% (26). Next, *Buteo buteo* (common buzzard) from France with a seroprevalence of 51% (27), *Ciconia ciconia* (white stork) from Portugal with a seroprevalence of 31.4% (28), *Pica pica* (Eurasian magpie) with 15.2% from Spain and *Anas indicus* (bar-headed goose) from Spanish zoos, with a seroprevalence of 7.1% (26, 29). In terms of molecular prevalence, in common buzzards from Turkey it was 92% (30), 18.75% in *Spatula clypeata* (northern shoveler) and in 6.25% *Anas crecca* (Eurasian teal) from Italy (31) and finally, merely 1.85% in white storks from Poland (32). Taken together, the published data shows that there is a significant divergence in infection prevalence, making any firm conclusions regarding the true epidemiological significance of birds in terms of toxoplasmosis difficult to reach. It is equally difficult to ascertain whether the frequency of infection correlates fully with diet alone. Perhaps this is due to relatively few individuals analyzed per species, as compared to the large set used by Wilson et al. (21). Indeed, the number of individuals of many wild species in a number of studies rarely exceeds 10, with 1–3 being most common. An inevitable limitation in terms of sampling wild birds is ‘convenience’ sampling of blood and tissues of game and birds in rehabilitation or rescue centers (such as in this study), and particularly in zoos, which may bias the results by over- and underestimating infection occurrence. Thus, even a relatively low occurrence of infection, 7.7% in owls as determined here, or the white storks from Poland (32), may confer a high capacity to transmit *T. gondii* to other species in environments in which there are large numbers of these birds. When considering human infection, wild game, such as pheasants and mallards, with a combined frequency of infection of 26% are relevant as reservoirs, however, it is unclear how probable or frequent infections actually are, as opposed to infections resulting from domestic species, like chickens, which are far more commonly consumed. Chickens are also efficient bioindicators for the contamination of the environment with oocysts, due to their feeding habits (pecking off the ground) (33). The occurrence of infection in chickens in Europe varies, with the greatest overall detected in backyard chickens 47% (34) in Germany and 41.2% in Greece (35), followed by free-range chickens, 3.2% in Greece (35), 5.6% in Portugal (36), and 18% in Ireland (37), and the lowest in caged chickens, 1.7% in Greece (35), 3.7% in Germany (34), and 0.4% in the Czech Republic, as expected (38). Interestingly, these studies show that even though free-range chickens are also exposed to the environment, and possibly oocysts, their seroprevalence is lower as compared to backyard chickens. The results of this study showed a statistically significant difference between the occurrences of infection in those from rural backyards, 80%, as opposed to 21% from peri-urban backyards (Table 4). However, given the relatively low number of samples analyzed here, it is unclear whether this variability in occurrence of infection is a general trend and how a country-wide pooled occurrence may compare to the published data. However, if variability of infection occurrence is inherent, a pooled prevalence in backyard chickens on a country-wide scale may not provide relevant information with respect to the true infection risk for humans. In terms of the significance of avians as reservoirs of infection for other animals, the key host to consider are cats (owned, feral, wild) which facilitate environmental transmission of *T. gondii*. It has been suggested that chickens are an efficient source of infection for cats (18), but it has also been estimated that cats kill anywhere from 136 million (Poland), 64.8 million (UK), and 1.73 million (Finland) wild birds annually and that ‘death by cat’ is the leading cause of mortality for garden birds in France and Belgium (39). With respect to domestic cats, which are most numerous worldwide, the above findings suggest that more relevant reservoirs of *T. gondii* may be wild birds living in cities and/or close proximity to urban areas. The molecular occurrence of infection between wild species residing in the urban, peri-urban and rural areas was not statistically different, 22.4, 27.3, and 22.7%, respectively (Table 4), suggesting equal opportunities for infection of other animals in these areas. The most numerous birds in the city (*n* = 79), all from the residential neighborhood of Surčin, were Corvidae (rooks and hooded crows) and Columbidae (wood pigeons), with a combined molecular occurrence of infection of 25.3%, indicating a fairly high probability for transmission. However, infection was detected in 15.6% of the wood pigeons, as opposed to 32.1% of rooks and 31.6% of hooded crows. As both Corvidae and Columbidae are suitable as cat prey, the results point to the conclusion that despite a high combined occurrence of infection, cats are far more likely to become infected when preying on Corvidae. To analyze potential animal and environmental *T. gondii* reservoirs in the residential neighborhood of Surčin, we collected rodents from local exterminators and Sava River water. Based on available information, the mice were collected from rodent traps which were set around small farms in close proximity to each other. The high molecular occurrence of infection (56.2%), may therefore be explained in part by the fact that mice live in groups on small territories, thus providing the opportunity for a relatively large number of them to become infected by a locally occurring source of *T. gondii* oocysts or tissue cysts. It stands to reason therefore that carnivorous or scavenging birds with habitats/feeding grounds within the mouse territory as well as cats living in the area have a fairly high probability of infection. *T. gondii* DNA was detected several years ago in the Danube River in water sampled in another residential neighborhood of Belgrade, Zemun (24). The locations on the Sava sampled here are on both banks of the river, several hundred meters apart. *T. gondii* DNA was detected at three locations of the four sampled. Although systematic sampling of the Sava and Danube in the Belgrade metropolitan area has not been done to date, the combined results point to the fact that oocyst contamination may be fairly high in some locations. The findings here point to a local source of oocysts, likely to be environmental rather than animal, as the sampling was done on different days. As much of the area of Surčin is within the river’s floodplain, the oocysts could have originated from the surrounding soil. Along with natural reservoirs, anthropogenic sources (waste) may also contribute to infection in omnivorous species and may in part explain the relatively high molecular occurrence of infection in hooded crows, rooks and mice. Based on the currently published data, including this study, the significance of avians in the epidemiology of toxoplasmosis is still unclear. It appears that species living in close proximity to humans in urban areas are more likely to be preyed upon by domestic cats (owned and feral), thus even a low prevalence of infection is sufficient to maintain the parasite in these environments. As far as parasite dissemination is concerned, it is not entirely clear whether migratory or sedentary birds have a greater capacity to spread *T. gondii*. Migratory birds may cover great distances, yet migrations occur twice annually, while some sedentary species, like rooks and wood pigeons, visit different feeding grounds on a daily basis. Due to the frequency of localized travel, sedentary birds may be more likely to facilitate spread on a smaller geographical scale. The city dwelling sedentary species analyzed here share their habitats/feeding grounds not only with cats but also other carnivorous wildlife, which increases the likelihood for transmission and spread of *T. gondii*. Our results further suggest that perhaps Corvids are important reservoirs of the parasite in urban environments due to a relatively high molecular occurrence of infection. Corvids may even be useful as bioindicators for the presence of *T. gondii* in urban environments, due to their population size and omnivorous feeding habits. Our findings did not support a strong dependency of the occurrence of infection on diet, when analyzed as a sole risk factor, possibly due to the low number of strictly carnivorous avian species (owls and raptors). Our analysis of the natural sources of infection however suggests that on a local scale, diet must be considered in context of the species ecology and habitat characteristics and availability of anthropogenic food sources in a particular area. Approximately half of the 11 species of city dwelling birds analyzed here originated from Surčin, which features a mosaic of natural wetlands, fishponds and forests along with agricultural plots, gardens and orchards which provide shelter, nesting and breeding habitats as well as a variety of food sources. Altogether, this resilient matrix of habitat and resources supports the increase in population size and biodiversity of avian species in close proximity to a highly urbanized area. With respect to *T. gondii*, this ecological setup facilitates environmental and host-to-host transmission and spread to other avian species, terrestrial wildlife and humans, thus feeding parasite strains through both the ‘wild’ and ‘domestic’ cycles. The importance of avian hosts in terms of the local population genetics of *T. gondii* remains to be investigated.

## Data Availability

The raw data supporting the conclusions of this article will be made available by the authors, without undue reservation.

## Footnotes

^1^QGIS.org, 2025.
